# The mutational analysis of mitochondrial DNA in maternal inheritance of polycystic ovarian syndrome

**DOI:** 10.3389/fendo.2023.1093353

**Published:** 2023-08-22

**Authors:** Shaheen Bibi, Ghulam Abbas, Muhammad Zahoor Khan, Tanzeela Nawaz, Qudrat Ullah, Aziz Uddin, Muhammad Fiaz Khan, Sajid Ul Ghafoor, Muhammad Shahid Nadeem, Sadia Tabassum, Muhammad Zahoor

**Affiliations:** ^1^ Department of Zoology, Hazara University, Mansehra, Pakistan; ^2^ Department of Biotechnology, University of Agriculture, Dera Ismail Khan, Pakistan; ^3^ Faculty of Veterinary and Animal Science, University of Agriculture, Dera Ismail Khan, Pakistan; ^4^ Department of Biotechnology and Genetic Engineering, Hazara University, Mansehra, Pakistan; ^5^ Department of Biochemistry, Faculty of Science, King Abdul-Aziz University, Jeddah, Saudi Arabia; ^6^ Department of Molecular Medicine, Institute of Basic Medical Sciences, University of Oslo, Oslo, Norway

**Keywords:** mitochondrial DNA, mutations, PCOS, genome, sequence analysis, pathogenicity

## Abstract

**Introduction:**

Polycystic Ovarian Syndrome (PCOS) is a globally prevalent condition that leads to infertility in women. While environmental factors contribute to PCOS, maternal genetics also play a significant role. Currently, there is no definitive test for identifying predisposition to PCOS. Hence, our objective is to discover novel maternal genetic risk factors for PCOS by investigating the genomes of patients from Pakistan.

**Methods:**

We utilized Next-Generation Sequencing (NGS) to sequence the complete mitochondrial DNA of three PCOS patients. Subsequently, we employed MitoTIP (Mitochondrial tRNA Informatics Predictor) and PON-mt-tRNA tools to identify variations in the mitochondrial DNA. Our analysis focused on the genes MT-RNR1, MT-RNR2, MT-ATP6, MT-TL2, and MT-CYTB, which displayed common variations in all three genomes. Additionally, we observed individual variations. The D-loop region exhibited the highest frequency of mutations, followed by the non-coding regions of RNR1 and RNR2 genes. Moreover, we detected frameshift mutations in the mitochondrially encoded NADH Dehydrogenase 2 (MT-ND2) and mitochondrially encoded NADH Dehydrogenase 5 (ND5) genes within individual genomes.

**Results:**

Our analysis unveiled six regions with common variations in the mitochondrial DNA of all three PCOS patients. Notably, the MT-RNR1, MT-RNR2, MT-ATP6, MT-TL2, and MT-CYTB genes exhibited these variations. Additionally, we identified individual variations in the mitochondrial DNA. The D-loop region displayed the highest mutation frequency, followed by the non-coding regions of RNR1 and RNR2 genes. Furthermore, frameshift mutations were detected in the MT-ND2 and ND5 genes within individual genomes.

**Conclusion:**

Through our study, we have identified variations in mitochondrial DNA that may be associated with the development of PCOS and have the potential to serve as predisposition tests. Our findings highlight the presence of novel mutations in the MT-RNR1, MT-RNR2, MT-ATP6, MT-TL2, and MT-CYTB genes, as well as frameshift mutations in the MT-ND2 and ND5 genes. Pathogenicity analysis indicated that most variants were likely to result in benign cysts. However, the frameshift mutations in the ND2 gene were associated with a high risk of complications and pathogenicity in PCOS. This is the first report identifying these mutations and their association with PCOS, contributing to our understanding of the genetic factors underlying the condition.

## Introduction

Polycystic ovarian syndrome (PCOS) is a complex endocrine disorder that affects up to 8%–13% of women in their reproductive age (1). PCOS is a growing concern worldwide, with increasing incidence rates reported (2). The disorder is characterized by the presence of numerous ovarian cysts visible through ultrasound inspection (3). PCOS is associated with various symptoms such as menstrual irregularities, hormonal dysfunction, dermatological issues, psychological problems, high cancer risk, and metabolic disorders (4). Various gynecological issues are associated with PCOS, including anovulatory infertility (5), variations in oocyte competency (OC), which can lead to subfertility (6, 7), endometrial dysfunction, and abnormal trophoblast invasion and placentation (8), which can increase the risk of miscarriage and pregnancy complications in women with PCOS (5). PCOS women who have hyperandrogenic conditions are also at a higher risk of developing pervasive developmental disorders (PDDs) (9). Hormonal dysfunction is considered a key feature of PCOS (8, 10), while insulin resistance and hyperandrogenism have also been reported, leading to decreased folliculogenesis and an increased risk of comorbidities and androgenic alopecia (11). The most prominent impact of PCOS on women’s lives is menstrual irregularities and ovarian cancer. PCOS is a multifactorial disorder, with several risk factors contributing to its etiology, including obesity, neuroendocrine status, environment or lifestyle, and genetic makeup (3). Variations in mitochondrial DNA (mtDNA) are increasingly recognized as a genetic cause (12, 13). Both nuclear and mitochondrial genetic variations have been associated with PCOS pathogenesis (14). Nuclear genes, including calpain 10 (CAPN10), cytochrome family P450, insulin (INS) gene, androgen receptor (AR), fat mass obesity (FTO) gene, and follicle-stimulating hormone receptor (FSHR) gene, have been shown to be associated with PCOS (15).

The mitochondrial genome is considered more vulnerable to oxidative damage and has a high mutation rate due to the lack of protective histones, inefficient DNA repair mechanisms, and its proximity to the electron transport chain (ETC), where oxygen-derived free radicals are frequently generated (16). Hence, the mitochondrial genome is considered a Pandora’s box of pathogenic mutations (16). This study was designed to screen the whole mitochondrial DNA (WMTDNA), comprising 37 mitochondrial genes using next-generation sequencing and to predict *in silico* the resultant common variations for pathogenicity. *In silico* analysis was used to evaluate the pathogenicity of mutations and their impact on subjects.

## Materials and methods

### Ethical statement

The experimental procedures were approved by the Ethical Committee of the Institution and Board of Advanced Studies and Research at Hazara University, Mansehra (21300), and Pakistan under notification number F.No.73/HU/ORIC/IBC/2017/400.

### Consent, recruitment of patients, and families

The Rotterdam criteria have been used for diagnosing PCOS patients (10). According to these criteria, a patient must have two of the following three symptoms: hyperandrogenism (biochemical or clinical), oligo- or anovulation, and polycystic ovary morphology (PCOM), as determined by ultrasound inspection.

**Figure 1:**
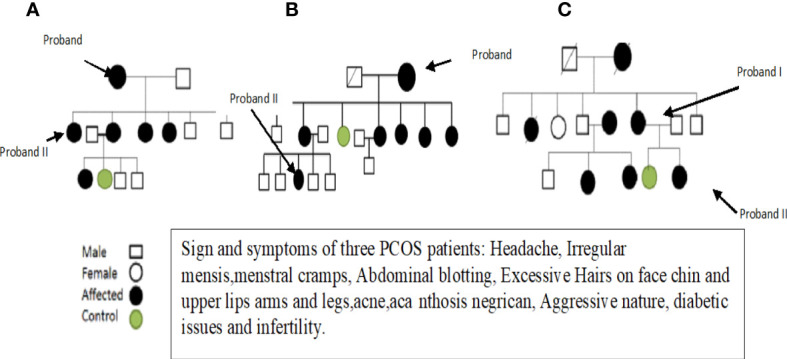
The family pedigree of patients 1 **(A)**, 2 **(B)**, and 3 **(C)** based on the information provided.

After obtaining informed consent and a physical examination by a gynecologist, selected patients were interviewed about their family history and other details, and pedigrees were constructed to trace the maternal inheritance pattern of their disorder. Saliva samples were then collected from each patient. Patients with hyperprolactinemia, thyroid and adrenal diseases, 21-hydroxylase deficiency, and androgen-secreting tumors were excluded because these disorders mimic the symptoms of PCOS. Finally, three fully expressed syndromic patients were selected and subjected to whole mitochondrial genome sequence (WMGS) analysis to draw the genetic portrait of mitochondrial mutational hotspots associated with maternally inherited PCOS. Variant calling and identification of homoplasmic and heteroplasmic mutations were carried out. After obtaining detailed family histories and information about deceased members, the pedigrees of the three families were constructed (see **Figure 1**). The mutations identified in WMGS were assessed in other family members of the probands through Sanger sequencing.

### DNA extraction, NGS analysis, and identification of variants

DNA was extracted from saliva samples using the phenol-chloroform method (17). Nanodrop quantification and gel electrophoresis were performed to determine the quantity and quality of the isolated DNA. Samples were then carefully labeled and stored at −20°C. The labeled samples were sent to a commercial company for DNA sequence analysis. Online DNA analysis tools like National Center for Biotechnology Information (NCBI) Blast, Universal Protein Resource (Uniprot), and UGENE were used to conduct further alignment and investigations. The resulting nucleotide sequences were compared to the revised Cambridge Reference Sequence (rCRS). We performed Sanger sequencing for validation of the identified common variations for D-loop, ATP6, MT-TL2, and CYTB.

In the UGENE (http://ugene.net/) editor, two nucleotide sequences were aligned, and variations were checked. Multiple sequence alignment was performed using integrated multiple sequence comparison by log expectations (MUSCLE) on UGENE.

### Score-based evaluation of tRNA variants for pathogenicity and validation by *in silico* predictive tools

Mitochondrial tRNA Informatics Predictor (MitoTIP) and PON-mt-tRNA were utilized to determine the pathogenicity of mitochondrial variations. MitoTIP was employed to assess the pathogenicity status of genetic variations, while the PON-mt-tRNA, a multifactorial probability-based prediction approach, was used to classify the studied and identified mitochondrial variants. Some variants were reported to be deleterious, some benign, one novel frameshift, and one pathogenic based on the results from both MitoTIP and PON-mt-tRNA. The difference in the degree of predicted variants is attributed to the fact that both tools operate on distinct algorithms/principles and consider diverse factors (18–20).

## Results

The current study focused on the mitochondrial genome of patients with maternally inherited PCOS. To achieve this, three patients were selected for the study, each from a different family with a history of maternally inherited PCOS. Furthermore, the experiment focused on analyzing the WMTDNA of these patients to identify any mutations or genetic variations that may be associated with the development of PCOS.

### Clinical evaluations

At the onset of the condition, the average age range was 30–35 years. Among these patients, two were unmarried and one was married but having infertility issues. One PCOS patient was suffering from diabetes due to insulin resistance, and the other two had catamenia, dermatological, metabolic, and hormonal issues. Other family members of these three patients also suffered from PCOS. Most of the symptoms, such as obesity, dermatological problems, catamenia, hirsutisms, and hormonal issues, are common in the three PCOS patients. The detailed clinical data of the PCOS patients are given in **Table 1**.

**Table 1:** Clinical manifestations of three PCOS patients selected for WMTDNA analysis.

Clinical characteristics	Patient 1	Patient 2	Patient 3
Remarks	Patient	Patient	Patient
Candidate’s age	30	28	35
Family history	+	+	+
Marriage history	Married	Unmarried	Unmarried
Catamenia problems	**+**	**+**	**+**
Irregular menses	**+**	**+**	**+**
Metrorrhagia	**+**	**+**	**+**
Amenorrhea	**+**	−	−
Diabetic problem	**+**	−	−
Insulin resistance	**+**	−	−
Dermatological problems	**+**	**+**	**+**
Hirsutism	**+**	**+**	**+**
Infertility problems	**+**	−	−
Obesity	**+**	**+**	**+**
Elevated abdominal circumference	**+**	**+**	**+**
Hormonal issues	**+**	**+**	**+**

**“+”** Presence of PCOS symptoms in PCOS patients; “−” Absence of PCOS symptoms in PCOS patients.

### Common mitochondrial DNA mutations were identified in the coding and noncoding region of the three PCOS patients in the present study

**Figure 2:**
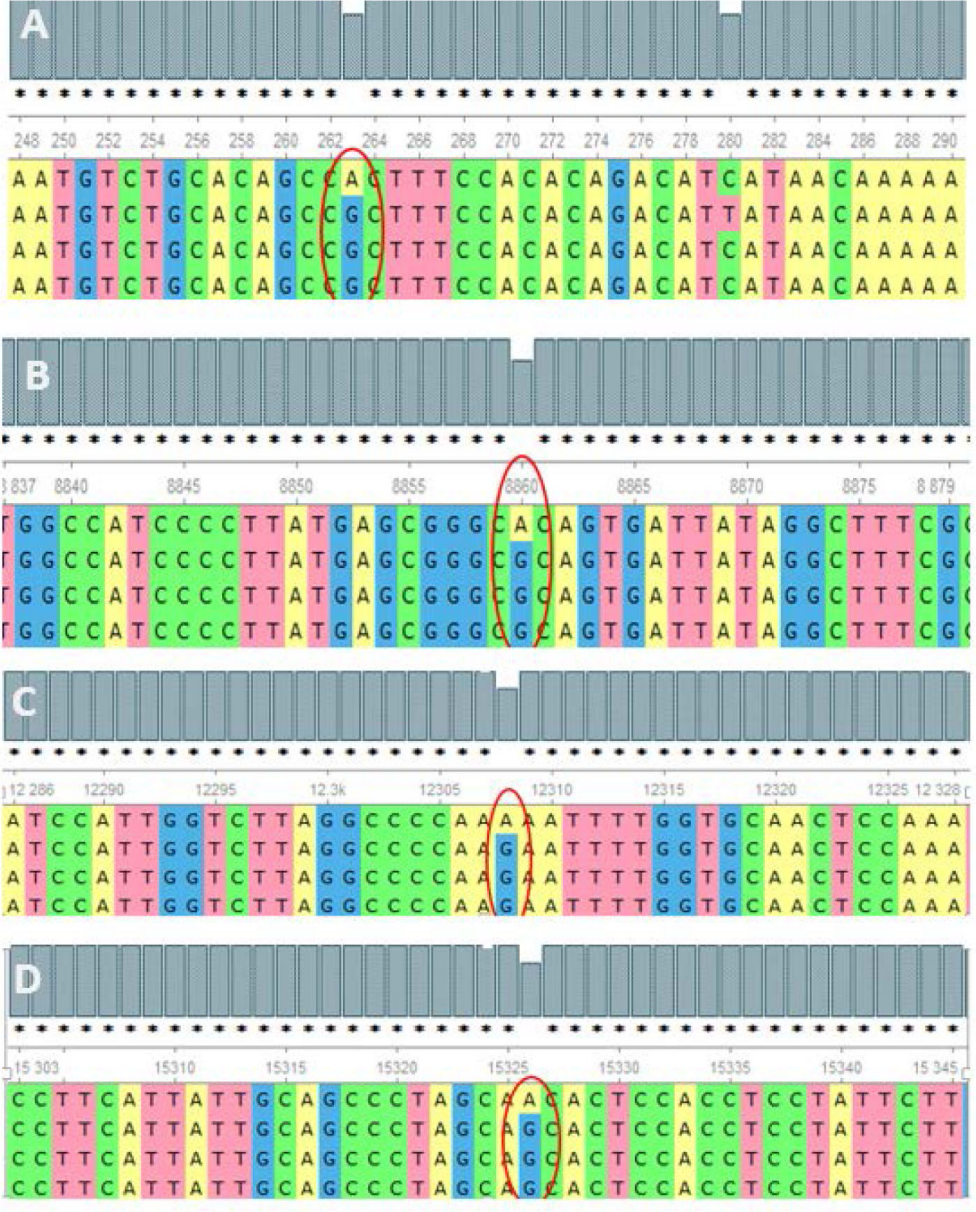
Alignment of the sequence resulted in nucleotides from maternally inherited PCOS-positive patients with rCRS Accession No. NC-012920.1 exhibiting mutations (encircled) at positions **(A)** 263A>G (D-loop), **(B)** 8860A>G (MT-ATP6), **(C)** 12308A>G (MT-TL2), and **(D)** 15326A>G MT-CYTB.

The analysis of the whole mitochondrial genome has enabled us to identify a set of mutations present in three familial subjects with PCOS. The mutations that were identified in all three genomes are presented in **Table 2**. Specifically, eight mutations at eight different positions in six genes were identified as common among the three PCOS patients. Of these, two mutations were located in the D-loop region, one in RNR1, one in RNR2, one in ATP6, one in MT-TL2, and one in CYTB, while four were missense variants, two were identified in intergenic regions, and one was a noncoding transcript. To further validate these findings, the mutations were revalidated in other PCOS patients through Sanger sequencing of the individual positions, as depicted in **Figure 2**.

**Table 2:** Common mitochondrial DNA mutations identified in the coding and noncoding regions of three PCOS patients in the present study.

Sr. No.	Mutation	Gene name	Complete name	Location
1	73A>G	D-loop	Displacement loop	Intergenic region
2	263A>G	D-loop	Displacement loop	Intergenic region
3	1438A>G	RNR1	12S ribosomal RNA	Noncoding transcript
4	3106CN>C	RNR2	16S ribosomal RNA	Missense variant
5	8860A>G	ATP6	ATP synthase F0 subunit 6	Missense variant
7	12308A>G	MT-TL2	Mitochondrial transfer RNA leucine 2	Missense variant
8	15326A>G	CYTB	Cytochrome *b*	Missense variant

### Mutations were identified in the whole mitochondrial genome sequence of the three PCOS patients in the present study

**Figure 3:**
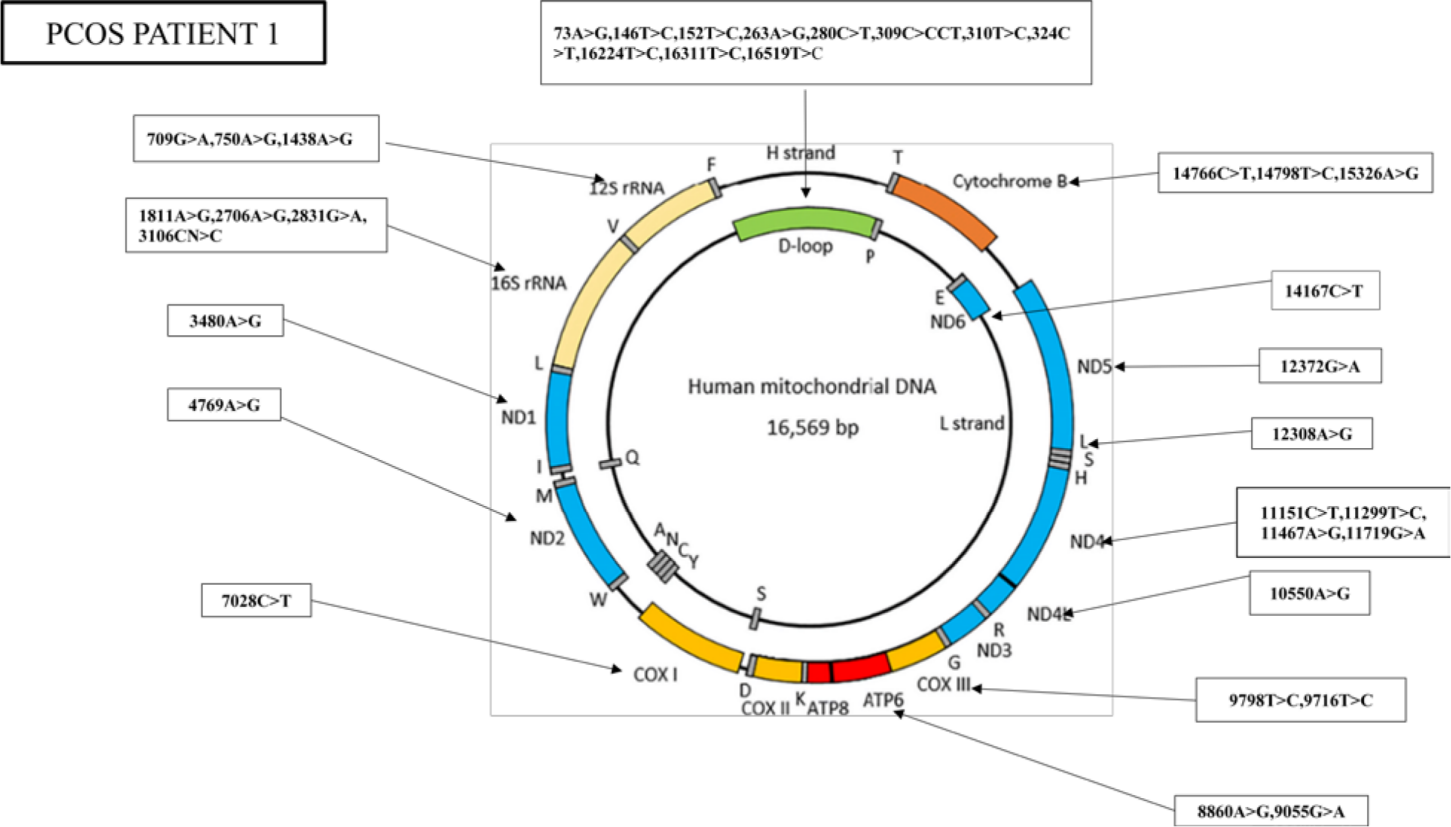
Whole mitogenome variants identified in PCOS patient 1. Mitochondria consist of two strands; an outer heavy strand and an inner light strand. Overall, 36 mutations were identified in the whole mitochondrial genome: 11 in D-loop, three in RNR1, four in RNR2, one in ND1, one in ND2, one in COX1, two in ATP6, two in −COX3, one in ND4L, four in ND4, one in ND5, one in ND6, and three in CYTB.

The nucleotide sequence analysis has identified 36 mutations in PCOS patient 1, including 11 variants in the D-loop region, seven variants in the *RNR1* and *RNR2* genes, nine synonymous mutations, and nine missense mutations (**Figure 3**).

**Figure 4:**
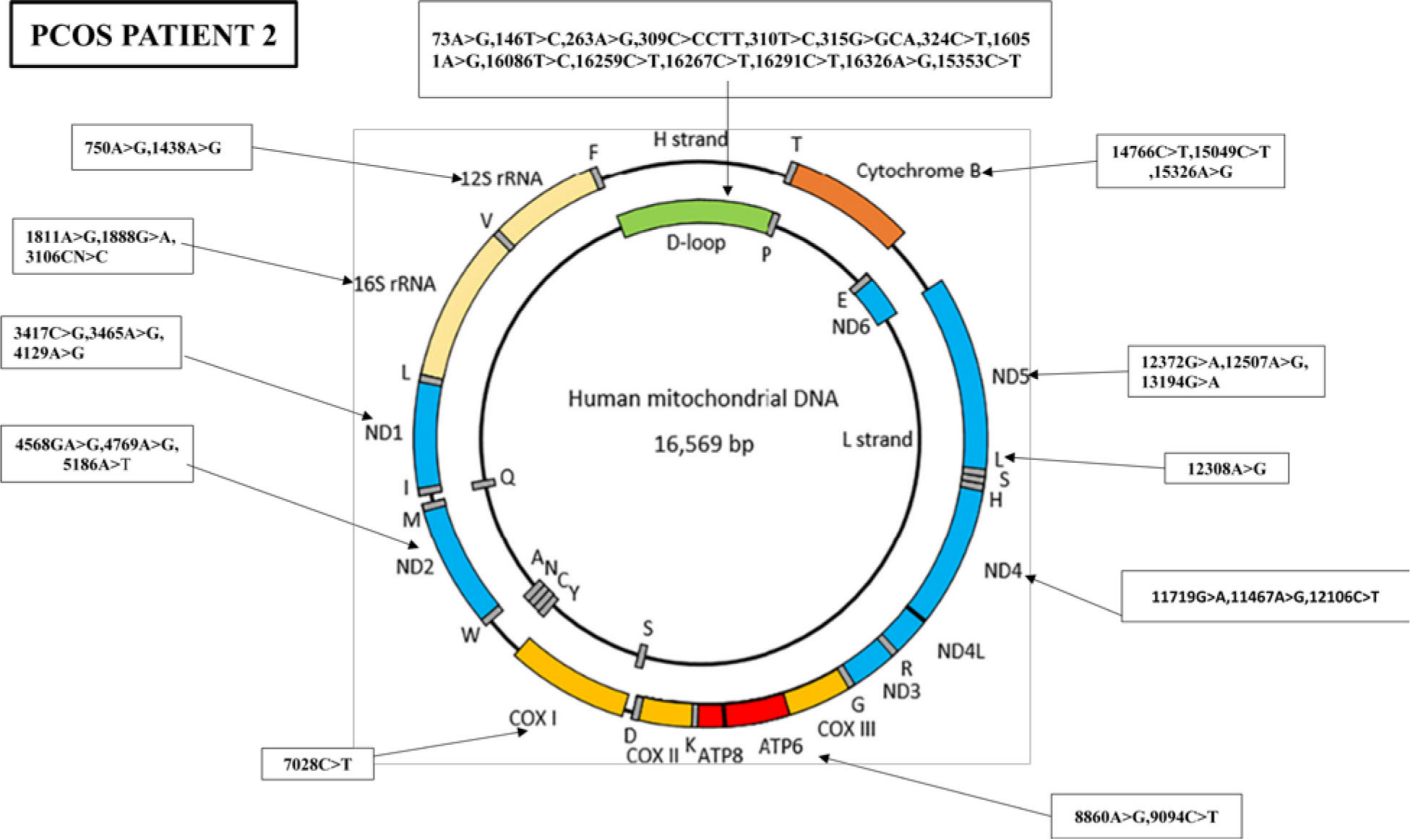
Mutations identified in the whole mitochondrial DNA sequence of PCOS-positive patient 2. Overall, 38 mutations were identified in the whole mitochondrial genome of PCOS patient 2: 14 mutations were found in D-loop, two in RNR1, three in RNR2, three in ND1, three in ND2, one in COX1, two in ATP6, three in ND4, three in ND5, one in MT-TL2, and three in CYTB.

Similarly, the nucleotide sequence analysis has identified 38 mutations in PCOS patient 2, including 14 variants in the D-loop region, five variants in the *RNR1* and *RNR2* genes, nine synonymous mutations and eight missense mutations, and one stop-loss and one frameshift (**Figure 4**).

**Figure 5:**
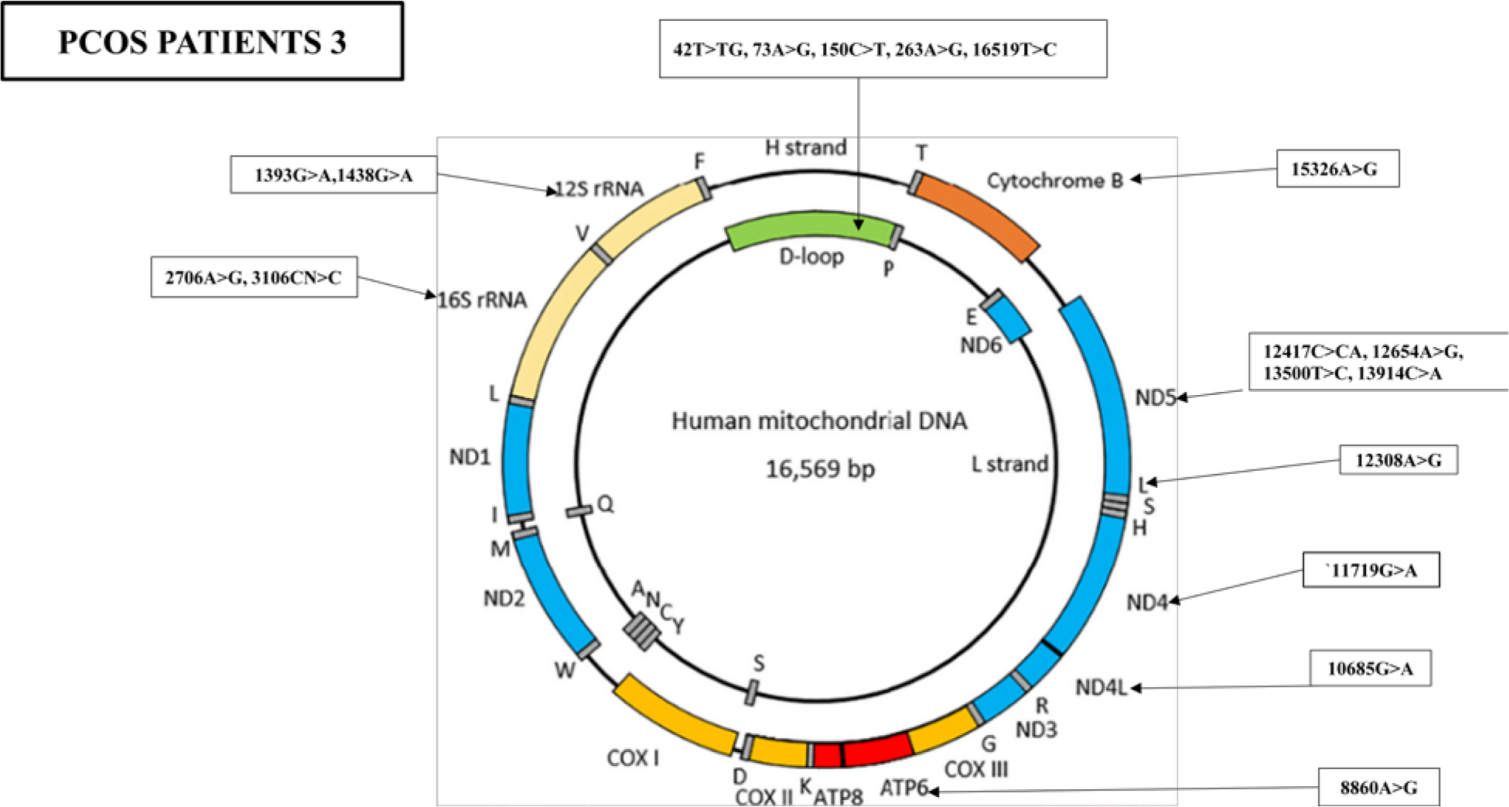
Mutations identified in the whole mitochondrial DNA sequence of PCOS patient 3. Overall, 19 mutations were identified in the whole mitochondrial genome: five mutations were found in D-loop, two variants in RNR1, two variants in RNR2, one in ATP6, one in ND4, one in ND4L, one in TL2, four in ND5, and one in CYTB.

Furthermore, the WMTDNA nucleotide sequence analysis has identified 19 mutations in PCOS-positive patient 3, including five variants in the D-loop region, four variants in the *RNR1* and *RNR2* gene, five synonymous mutations and three missense mutations, and one stop-loss and one frameshift (**Figure 5**).

### Pathogenicity status of the studied mutations

The pathogenicity of the identified variants was evaluated using different tools. Results revealed that among all the common mutations identified in the three whole mitogenomes of PCOS patients after NGS analysis, mutations reported in RNR1 at positions 709A>G, 750A>G, 1438A>G, and 1393 G>A were found to be resulting in translation defects that were pathogenic. MT-12S rRNA variations are possibly associated with disruption of mitochondrial function. The mutations found in RNR2 at positions 2706A>G, 2831G>A, 3106CN>C, and 1888G>A. These mutations are present at the 530 loops of the ribosome, and they affect the codon–anticodon interaction at the A site (acceptor site). These mutations lead to the improper movement of the small subunit (SSU) head during translocation and finally the translocation (**Table 3**).

**Table 3:** Pathogenicity prediction of genetic variants detected in PCOS patients.

Sr. No.	Genes	Mutation	rsID	SIFT; PolyPhen	MitoTIP score	Pon-mt-tRNA	Comment
1	MT-RNR1	1438A>G	rs2001030	–	–	–	Noncoding transcript exon variant
2	MT-RNR2	3106CN>C	rs368669629	–	–	–	Noncoding transcript exon variant
3	MT-CYTB	15326A>G	rs2853508	0.28 tolerated; 0.009 benign	–	–	
4		11719 G>A					Synonymous variant
5	MT-ATP6	8860A>G	rs2001031	0.1; 0.003 tolerated	–	–	Missense variant
6	MT-TL2	12308A>G	rs2853498	–	11.8509 (Q3)	0.41	

### ML probability of pathogenicity: average probability of pathogenicity predicted by 20 machine learning (random forests) predictors

In addition, a mutation in the *MT-ATP6* gene at position 8860A>G with a score of 0.1 was declared as tolerated with a score of 0.003. A mutation in mitochondrial transfer RNA leucine 2 at position 12308A>G with a score of 11.8509 was declared likely neutral with a score of 0.41. A mutation at position 11719G>A in the *ND4* gene is a synonymous variant and pose no impact on the amino acid type.

## Discussion

Overall, the present study aimed to investigate the mitochondrial genome of patients with maternally inherited PCOS. Three patients, each from a different family with a history of maternally inherited PCOS, were selected for the study. The study focused on analyzing the WMTDNA of these patients to identify any mutations or genetic variations that could be associated with the development of PCOS. By analyzing the mitochondrial genome of these patients, we aimed to gain insights into the potential genetic factors that contribute to the development of this disorder, specifically those that are passed down maternally.

PCOS is a common endocrine disorder that affects reproductive-age women worldwide and is the leading cause of ovulatory dysfunction and infertility (21). PCOS is characterized by hyperandrogenism, anovulation, and polycystic ovaries, along with insulin resistance, obesity, and metabolic disorders (1). Several predisposing risk factors, including genetic, neuroendocrine, lifestyle/environmental, and obesity, have been linked to PCOS development (3). Moreover, mtDNA mutations are also considered to contribute to the pathogenesis of PCOS. MI mutations that were potentially associated with PCOS-IR were as follows: mt-tRNA^Leu(UUR)^ A3302G and C3275A mutations; mt-tRNA^Gln^ T4363C and T4395C mutations; mt-tRNA^Ser(UCN)^ C7492T mutation; mt-tRNA^Asp^ A7543G mutation, mt-tRNA^Lys^ A8343G mutation, mt-tRNA^Arg^ T10454C mutation, and mt-tRNA^Glu^ A14693G mutation (22). They utilized a whole mitochondrial genome sequencing analysis for three maternally inherited PCOS familial subjects. Their findings revealed several variations in mtDNA. Patient 1 had 11 variants in the d-loop region, including 73A>G, 146T>C, 152T>C, 263A>G, 280C>T, 309C>CCT, 310T>C, 324C>T, 16224T>C, 16311T>C, and 16519T>C. The variant 73A>G, 146T>C, and 152T>C have been previously reported to be associated with gastric colon and oral cancer, PCOS, and breast cancer (23–25). Women with PCOS may be more susceptible to some cancers due to their abnormal metabolic and hormonal conditions.

Prolonged hormone stimulation is known to be associated with the development of endometrial, ovarian, and breast cancers in women (24, 26). In this study, we identified several mtDNA variants in the three maternally inherited PCOS familial subjects that have been previously reported to be associated with various types of cancer and other diseases. Variant 263A>G was found to be linked with perilesional skin and skin tumors and PCOS (27). Variants 310T>C and 324C>T have been associated with epithelial ovarian cancer (28), while 16224T>C and 16311T>C have been linked to perilesional skin and skin tumors (27). Variant 309C>CCT has been associated with the etiology of malignant melanoma (29). One variant in the *COX1* gene, which has been reported to be associated with PCOS, esophageal cancer, congenital contract, and obesity, was found in patient 1 (23, 30, 31). Similarly, variant 11719G>A has been linked to breast cancer, obesity, and PCOS (24, 31). Moreover, one variant reported in transfer RNA leucine 2 at nucleotide position 12308A>G has been associated with breast cancer, colorectal cancer, prostate and kidney cancer, Alzheimer’s disease, and cardiomyopathy (24, 25, 32, 33).

Our investigation identified three variants in the *CYTB* gene, including 14766C>T and 14798T>C, which have not been previously reported in association with PCOS (27). Women with a predisposition to PCOS may experience metabolic and hormonal issues such as insulin resistance and hyperandrogenism, which can lead to weight gain and eventually obesity. Obesity, in turn, can exacerbate the symptoms of PCOS, resulting in further metabolic complications and reproductive abnormalities. Our study also identified two mitochondrial variants in the *ATP6* gene, namely 8860A>G and 9055G>A. These variants have been previously associated with hypertrophic cardiomyopathy (25) and nonsyndromic hearing loss (28). The vascular characteristics of arterial walls involved in the atherogenic process may be directly influenced by androgen excess in PCOS, as reported by Wu et al. (34) in 2020. Furthermore, mutations were discovered in both the coding and noncoding regions of mitochondria in selected patients who suffered from various issues such as skin problems, hormonal imbalances, diabetes, menstrual problems, and infertility. Other family members were also identified as having this disorder (**Table 1**).

Fourteen variants were identified in the D-loop of patient 2, including 73A>G, previously found to be associated with gastric colon and oral cancer (24), 146T>C with oral cancer (23) and PCOS, 263A>G in association with PCOS (24), 310T>C with malignant melanoma (26), and 513G>GCA with nodular sclerosing Hodgkin lymphoma (Mitomap). Variants not previously reported in the literature with any disease but identified in our study that may be associated with PCOS are 16051A>G, 16086T>C, 16259C>A, 16267C>T, 16291C>T, 16326A>G, and 16353C>T.

In PCOS patient 2, two variants were identified in RNR1, including 750A>G, which has been reported to be associated with brain tumors (28), obesity (31), and PCOS (24), while 1438A>G is associated with obesity (31), type 2 diabetes, Parkinson’s disease, and PCOS. In RNR2, three variants were reported, including 1811A>G, which has been associated with congenital cataracts (30) and PCOS (24). However, 1888G>A and 3106CN>C have not been reported in previous literature with any disease. Three variants were identified in ND1, including 3417C>G, 3465A>G, and 4129A>G, with no previous reports in association with any disorder, making them novel findings in this investigation. Similarly, three variants were reported in ND2, including the 4769A>G (frameshift) variant previously associated with esophageal cancer and PCOS (24), while 4569GA>G and 5186A>T (stop loss) variants have not been reported in previous studies with any disease. In the *ATP6* gene, two variants were identified at position 8860A>G, reported to be associated with hypertrophic cardiomyopathy and PCOS, while 9094C>T is associated with primary ovarian insufficiency (35). In PCOS patient 2, a single variant was found in transfer RNA leucine 2, previously associated with breast cancer (24), and colorectal and kidney cancer (33). Three variants were identified in the cytochrome *b* gene, including 15326A>G, previously associated with PCOS (27), while 14766C>T and 15049C>T mutations have not been reported in any previous studies.

Five variants were identified in the mitochondrial D-loop region of PCOS patient 3, including 73A>G, 150C>T, 263A>G, and 16519T>C, previously associated with PCOS and gastric, colon, and oral cancers. In addition, 42T>TG was also identified. Two variants were identified in RNR1, including 1393G>A with no previous reports and 1438A>G with a reported association with obesity (31), type 2 diabetes, Parkinson’s disease, and PCOS (27). However, 3106CN>C has not been reported in previous literature with any disease.

One variant was identified in ND4L at position 10685G>A, and four variants were identified in ND5 at positions 12417C>CA, 12654A>G, 13500T>C, and 13914C>A. One variant was identified in ND6 at position 14305G>A, and one variant was identified at position 15326A>G, both associated with PCOS (27). We identified mutations in both the coding and noncoding regions of mitochondria, which may have phenotypic effects on patients. PCOS patient 3 also suffered from skin issues such as acne and hirsutism, hormonal problems, diabetes, menstrual problems, frequent urination, and some psychological issues. Other family members were also found to be suffering from this disorder (refer to **Table 1**). The identified mutations and their biological effects in our current study and in other previous studies have been summarized in **Table 4**.

**Table 4:** Mitochondrial DNA mutations identified in our stud of the WMTDNA of PCOS patients and their association with other diseases.

Sr. No.	Mutation	Gene name	Biological effect in the current study	Biological function	References
1	73A>G	D-loop	PCOS	Breast cancer; PCOS; gastric, colon, and oral cancers	Czarnecka et al. (24) and Zhuo et al. (27)
2	263A>G	D-loop	PCOS	Perilesional skin and skin tumor, PCOS	Zhuo et al. (27) and Durham et al. (36)
3	1438A>G	MT-RNR1	PCOS	PCOS	Zhuo et al. (27) and Wang et al. (31)
4	3106CN>C	MT-RNR2	PCOS, dermatological issues	PCOS, dermatological issues	In the current study
5	8860A>G	MT-ATP6	PCOS	Hypertrophic cardiomyopathy, PCOS	Zhuo et al. (27) and Grasso et al. (32)
6	11719G>A	MT-ND4	PCOS	Breast cancer, obesity, PCOS	Czarnecka et al. (24) and Zhuo et al. (27)
7	12308A>G	MT-TL2	PCOS	Breast, colorectal, and kidney cancers; Alzheimer’s disease; cardiomyopathy	Czarnecka et al. (24), Weigl et al. (37), and Booker et al. (33)
8	15326A>G	MT-CYTB	PCOS	PCOS	Zhuo et al. (27)

Eight different mutations at a different positions in six genes, these mutations are already reported in association with different diseases.

## Conclusions

In conclusion, studying the mutation spectrum of the entire mitochondrial genome is a valuable tool for investigating various maternally inherited genetic disorders in humans, including PCOS. Mitochondrial dynamics, including the mutational spectrum, can be explored to elucidate the etiology of such genetic diseases, given that mitochondria have all the mechanisms for energy transduction in many cells and organs. Homoplasmic variations can have catastrophic consequences, while heteroplasmic mutations may have a lesser impact. This study identified mutations in the *D-loop*, *MT-RNR1*, *MT-RNR2*, *MT-ATP6*, *MT-TL2*, and *MT-CYTB* genes that are most likely associated with the etiology of maternally inherited PCOS in Pakistan. To help patients receive appropriate treatment, genetic testing for the condition and public awareness efforts should be implemented. To validate the association of these mutations with PCOS, it is recommended to predict the defects and replicate the sequence analysis with a larger sample size in other parts of the world. Furthermore, the paternal inheritance pattern should be studied in PCOS patients from Pakistani families. Overall, this study highlights the potential of mitochondrial genetic variations as a novel biomarker for PCOS diagnosis and management. Further research is needed to establish a causal relationship between these mutations and the development of PCOS.

## Data availability statement

The original contributions presented in the study are included in the article/supplementary material. Further inquiries can be directed to the corresponding authors.

## Ethics statement

The studies involving human participants were reviewed and approved by Ethical Committee of the Institution and Board of Advanced Studies and Research at Hazara University, Mansehra (21300), Pakistan under notification numberF.No.73/HU/ORIC/IBC/2017/400. The patients/participants provided their written informed consent to participate in this study.

## Author contributions

SB, MK, GA, ST, and MZ designed the study and wrote the manuscript. ST and MZ supervised the manuscript. SB, TN, QU, MK, AU, MF, SG, MN, and GA helped in the collection of data resources and editing of the final version of the manuscript. Moreover, data analysis and collection were completed by SB, TN, and MF. All authors contributed to the article and approved the submitted version.
